# Determinants of post-malarial anemia in African children treated with parenteral artesunate

**DOI:** 10.1038/s41598-019-54639-4

**Published:** 2019-12-02

**Authors:** Katrin Scheu, Ayola Akim Adegnika, Marylyn M. Addo, Daniel Ansong, Jakob P. Cramer, Svenja Fürst, Peter G. Kremsner, Florian Kurth, Thomas Jacobs, Jürgen May, Michael Ramharter, Justice Sylverken, Christof D. Vinnemeier, Tsiri Agbenyega, Thierry Rolling

**Affiliations:** 1 Division of Infectious Diseases, I. Department of Medicine, University Medical Center Hamburg-Eppendorf, Hamburg, Germany; 2 German Center for Infection Research (DZIF), Hamburg-Lübeck-Borstel-Riems, Germany; 3 Centre de Recherches Médicales de Lambaréné, Lambaréné, Gabon; 4 Institute of Tropical Medicine, University Medical Center Tübingen, Tübingen, Germany; 5 Central African Network for Tuberculosis, Aids and Malaria (CANTAM), Brazzaville, Republic of Congo; 6 Department of Clinical Immunology of Infectious Diseases, Bernhard Nocht Institute for Tropical Medicine, Hamburg, Germany; 7 School of Medical Sciences, Kwame Nkrumah University of Science and Technology, Kumasi, Ghana; 8 Department of Tropical Medicine, Bernhard Nocht Institute for Tropical Medicine & I. Department of Medicine, University Medical Center Hamburg-Eppendorf, Hamburg, Germany; 9 Medizinische Klinik mit Schwerpunkt Infektiologie und Pneumologie, Charité Universitätsmedizin Berlin, Berlin, Germany; 10 Protozoa Immunology, Bernhard-Nocht-Institute for Tropical Medicine, Hamburg, Germany; 11 Department of Infectious Diseases Epidemiology, Bernhard-Nocht-Institute for Tropical Medicine, Hamburg, Germany; 12 Present Address: Infectious Disease Service, Memorial Sloan Kettering Cancer Center, New York, NY USA; 13 Present Address: Coalition for Epidemic Preparedness Innovations (CEPI), London, UK

**Keywords:** Paediatric research, Malaria

## Abstract

The pathophysiology of malarial anemia is multifactorial and incompletely understood. We assessed mechanistic and risk factors for post-malarial anemia in Ghanaian and Gabonese children with severe *P*. *falciparum* malaria treated with parenteral artesunate followed by an oral artemisinin-combination therapy. We analyzed data from two independent studies in which children were followed on Days 7,14, and 28 after treatment with artesunate. Specific hematological parameters included the presence of hemoglobinopathies and erythropoietin. Presence of once-infected erythrocytes was assessed by flow cytometry in a sub-population. Of 143 children with a geometric mean parasitemia of 116,294/µL (95% CI: 95,574–141,505), 91 (88%) had anemia (Hb < 10 g/dL) at presentation. Hemoglobin increased after Day 7 correlating with increased erythropoiesis through adequate erythropoietin stimulation. 22 children (24%) remained anemic until Day 28. Post-artesunate delayed hemolysis was detected in 7 children (5%) with only minor differences in the dynamics of once-infected erythrocytes. Hyperparasitemia and hemoglobin at presentation were associated with anemia on Day 14. On Day 28 only lower hemoglobin at presentation was associated with anemia. Most children showed an adequate erythropoiesis and recovered from anemia within one month. Post-artesunate delayed hemolysis (PADH) and hyperparasitemia are associated with early malarial anemia and pre-existing anemia is the main determinant for prolonged anemia.

## Introduction

The superior efficacy of artesunate over quinine in the treatment of severe malaria has been shown in different clinical and geographical settings^1–4^. Its use is not restricted by adverse events typical of quinine, such as QT prolongation, visual and auditory disturbances, and severe hypoglycemia^5^. Consequently parenteral artesunate is recommended as the treatment of choice for severe malaria by the World Health Organization^6^. The clinically most relevant adverse event of parenteral artesunate is delayed hemolysis occurring approximately two weeks after treatment^7,8^. Hemolysis affects between 20 and 30% of adult travelers from Europe with no or waning semi-immunity against malaria and high parasitemia treated with artesunate^9^. Delayed hemolysis has been reported in 5% of African children exposed to artesunate^10,11^.

Anemia is highly prevalent in African children under the age of 5 years^12^. The causes of anemia in this population are manifold and range amongst others from iron-deficiency to parasitic infections and genetic traits^12,13^. Additionally, anemia is major clinical hallmark of malaria and related to direct destruction of red blood cells by the parasite but also indirect effects, such as disruption of erythropoiesis and immune mechanisms^14^. Here we describe the recovery from malarial anemia in children with severe malaria treated with artesunate and assess predictive and mechanistic factors contributing to prolonged anemia.

## Material and Methods

### Patient population

This analysis is based on individual patient data from two studies: between April and September 2012 patients were recruited into a sub-study of the “Comparative, Open Label, Dose and Regimen Optimization Follow-up Study of Intravenous and Intramuscular Artesunate in African Children with Severe Malaria” (PACTR201102000277177) at the Komfo Anokye Teaching Hospital in Kumasi, Ghana, and at the Centre de Recherches Médicales de Lambaréné, Gabon, conducted by the Severe Malaria in African Children Network (SMAC)^15,16^. The primary endpoint of this study was the incidence of post-artesunate delayed hemolysis. Exploratory results of this sub-study have been published^10^.

Between January and August 2015 patients were recruited into a second study at the St. Michael’s Hospital in Pramso and the Komfo Anokye Teaching Hospital in Kumasi, Ghana.

### Study procedures

Children aged 6 months to 10 years were included in both studies if they presented to a study site with a primary diagnosis of *P*. *falciparum* malaria (>5000 parasites/µL on a thick blood smear) with signs or symptoms requiring hospitalization as judged by the treating physician as per the definitions of the Severe Malaria in African Children (SMAC) network reflecting standard practice in most African settings^17,18^. In both studies, patients were treated with parenteral artesunate followed by a course of weight-adapted oral artemether/lumefantrine. Within the first study, all patients received a total dose of 12 mg/kg artesunate according to three randomly assigned regimens (3 doses of 4 mg/kg body weight intravenously, 3 doses of 4 mg/kg body weight intramuscularly, or 5 doses of 2.4 mg/kg body weight intramuscularly). Within the second study, patients received at least three weight-adapted doses of parenteral (i.m. or i.v.) artesunate (2.4 mg/kg). If the patient was able to tolerate oral medication, treatment was switched to artemether/lumefantrine at this time point – else parenteral artesunate was continued. In both studies patients received folic acid supplementation for at least 14 days after treatment initiation.

Procedures have been described before^10^. Visits were scheduled on Day 0 (first day of treatment), Days 7 (±2), 14 (±2), and 28 (±4). The second study included an additional visit on Day 2.

Reticulocytes were assessed microscopically: after supravital staining with brilliant cresyl blue, reticulocytes per 1,000 erythrocytes were counted. Haptoglobin and erythropoietin was measured from serum stored at −80 °C after the end of recruitment by a commercial quality-controlled laboratory in Hamburg, Germany.

Genotyping of red cell polymorphisms was performed by high-throughput genotyping using fluorescent melting curve assays on 384-well microplate formats in a homogenous system (LightTyper, Roche Diagnostics)^10,19,20^.

Samples from the second study were assessed for the presence of once-infected (“pitted”) erythrocytes^21,22^. Erythrocytes were analyzed by flow cytometry (FACSCalibur™, BD Biosciences). Erythrocytes were incubated with monoclonal mouse anti-RESA (ring-infected erythrocyte surface antigen) antibodies (mAb 28/2, Walter + Eliza Hall Institute of Medical Research, Australia) followed by Peridinin chlorophyll protein (PerCP)-labelled anti-mouse-IgG to label RESA-positive erythrocytes. Nucleic acid staining was performed with Syto 16 (life technologies™). RBCs were plotted in two-dimensional scattergrams and gated according to their logarithmic forward and side scatter properties. Syto16 (DNA staining) was detected in the FL1 channel, PerCP (RESA-staining) was detected in the FL3 channel.

Double-positive (PerCP and Syto 16) erythrocytes were defined as infected, PerCP-positive, SYTO 16-negative erythrocytes as once-infected erythrocytes. Flow-cytometry data analyses was performed using FlowJo v10 software (Tree Star, Inc. Ashland, OR, USA). Two representative plots of one patient showing the switch from infected to once-infected RBCs are available as Supplementary Fig. 1.

### Definition of anemia

We defined anemia as a hemoglobin level lower than 10 g/dL, corresponding to at least a moderate anemia as defined by the World Health Organization^23,24^.

### Definition of post-artesunate delayed hemolysis (PADH)

PADH was defined as previously reported by our group^10^. Patients had to present both any decrease in hemoglobin and any increase in LDH between Days 7 (±2) and 14 (±2) in combination with both an elevated LDH (>350 IU/L) and low haptoglobin (<0.3 mg/dL) on Day 14.

### Statistical analyses

Statistical analyses were performed with Stata IC 15 (StataCorp, College Station, TX, USA). Descriptive characteristics were reported using absolute and relative frequency for categorical variables, mean and 95% confidence interval for normally distributed, and median and interquartile range for non-normally distributed continuous variables. Continuous variables were represented by their mean and standard error of the mean in figures. The association between anemia level and transfusion status was assessed by a chi-square test. The difference in once-infected pitted erythrocytes between patients with PADH and those without was tested by the Mann-Whitney-U-test. To build models predicting anemia on Day 14 (±2) and 28 (±4) variables which were associated in the univariable analysis with anemia at a p-level of 0.1 were included in a multivariable logistic regression model.

### Ethical considerations

Study protocols have been reviewed and approved by the respective institutional review boards (Committee on Human Research Publication and Ethics of Medical Sciences, Kwame Nkrumah University of Science and Technology, Kumasi, Ghana, and the institutional review board of the Centre de Recherches Médicales in Lambaréné, Gabon). Children were only included if informed consent was provided by the parent or legal guardian. All study procedures were performed in concordance with the Declaration of Helsinki.

## Results

**Figure 1:**
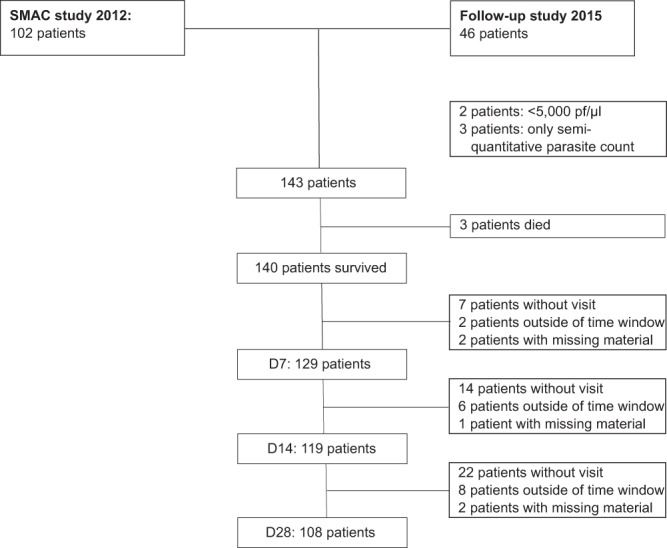
Patient diagram of the analysis population.

143 patients were eligible for this analysis, as represented in the patient flow diagram (Fig. 1). Baseline characteristics of the study population are described in Table 1. Children had a median age of 3.3 years (IQR: 2–5.5) and 88 (62%) were boys (Table 1). Geometric mean parasite density at presentation was 116,294/µL (95% CI: 95,574–141,505). 83 children (58%) fulfilled WHO criteria for complicated malaria, the most common being prostration (n = 69), hyperparasitemia (>200,000/µL; n = 47), and jaundice (n = 19). Three children died during the follow up period due to severe malaria.

**Table 1:** Baseline characteristics of children at presentation.

Variable	Total (n = 143)	Ghana (n = 96)	Gabon (n = 47)
Female Sex, n (%)	55 (38%)	40 (42%)	15 (32%)
Median Age, years (IQR)	3.3 (2.0–5.5)	3.2 (2.0–5.1)	4.0 (2.0–5.6)
Mean weight, kg (95% CI)	14 (13–15)	14 (13–15)	15 (13–17)
Mean height, cm (95% CI)	98 (94–101)	95 (91–99)	103 (96–111)
Mean temperature at admission (95% CI)	38.2 (38.0–38.4)	38.2 (38.0–38.4)	38.3 (38.0–38.6)
Hb Day 0, g/dL (95% CI)	8.7 (8.3–9.1)	8.6 (8.1–9.2)	8.8 (8.3–9.3)
RBC Day 0, ×10^6^/µL (95% CI)	3.6 (3.4–3.7)	3.4 (3.2–3.6)	3.9 (3.6–4.1)
WBC Day 0, ×10^3^/µL (95% CI)	9.9 (9.3–10.6)	10.1 (9.3–10.9)	9.6 (8.5–10.7)
Platelets Day 0, ×10^3^/µL (95% CI)	103 (90–116)	90 (76–103)	130 (103–158)
Geometric mean parasite density, /µL (95% CI)	116,294 (95,574–141,505)	140,912 (111,933–177,391)	78,565 (55,174–111,871)
Complicated malaria, n (%)	83 (58%)	69 (72%)	14 (30%)
- Cerebral malaria	− 19	− 5	− 14
- Repeated seizures	− 15	− 14	− 1
- Severe anemia (≤5 g/dL)	− 16	− 15	− 1
- Shock	− 3	− 3	− 0
- Hyperparasitemia (≥200,000/µL)	− 47	− 37	− 10
- Hypoglycaemia	− 10	− 6	− 4
- Jaundice	− 19	− 15	− 4
- Acute renal failure	− 1	− 1	− 0
- Significant spontaneous bleeding	− 2	− 2	− 0
- Prostration	− 69	− 65	− 4

**Figure 2:**
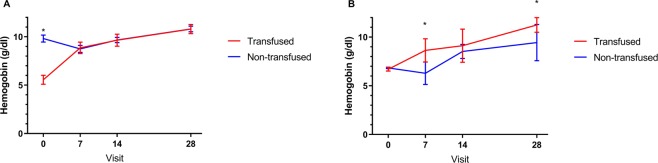
Hemoglobin values over time stratified by transfusion status (mean ± SEM) (**A**) all children. (**B**) Children with hemoglobin values between 6 g/dL and 7 g/dL at presentation *p < 0.05.

Upon presentation, 91 children (64%) were anemic (Hb <10 g/dL). 37 children (26%) with a mean hemoglobin of 5.5 g/dL (95% CI 5.1–6.0) received a blood transfusion. Children without transfusion had a mean hemoglobin of 9.8 g/dL (95% CI: 9.4–10.2). Figure 2A depicts the hemoglobin course over time in all children with data available: mean hemoglobin levels increased from Day 7 onwards and did not differ between patients who had and who had not received a transfusion. Highest hemoglobin levels were seen on Day 28 with a mean value of 10.8 g/dL (95% CI: 10.5–11.1) in those without a transfusion and of 10.7 g/dL (95% CI: 10.3–11.3) in those who had received a transfusion.

All patients with a hemoglobin lower than 5 g/dl at presentation received a transfusion, while no patient with a hemoglobin equal or higher than 9 g/dl received a transfusion. Transfusion rates gradually decreased between these values (88% for Hb ≥5 g/dL and <6 g/dL; 59% for Hb ≥6 g/dL and <7 g/dL; 20% for Hb ≥7 g/dL and <8 g/dL; 5% for Hb ≥8 g/dL and <9 g/dL). To explore the impact of transfusion on hemoglobin during follow-up, a restricted analysis was performed in children with hemoglobin levels between 6 g/dL and 7 g/dL (n = 13 transfused children and 19 non-transfused children). This restriction was chosen for three reasons: (a) to match hemoglobin levels at presentation; (b) as in this group there was the largest variability in transfusion patterns; (c) transfusions are not generally recommended over 7 g/dL. Children who received a transfusion had higher hemoglobin levels at follow-up with a mean hemoglobin at Day 28 of 11.2 g/dL (95% CI: 10.5–12.0) compared to those without a transfusion (9.4 g/dL; 95% CI: 7.6–11.3; Fig. 2B).

To assess risk factors of prolonged anemia on Day 14 and 28, patients who received a transfusion were not further considered (Table 2). After adjusting for hyperparasitemia and age, hemoglobin at presentation was associated with anemia on Day 14 (adjusted odds ratio 0.27 (0.16–0.47) for each unit increase in hemoglobin; n = 92). Children with hyperparasitemia had 8.76 (1.64–46.82; p = 0.011) higher odds of anemia on day 14 than those without hyperparasitemia, while age was not associated with anemia on day 14 in the multivariable analysis. Only hemoglobin at presentation remained predictive for anemia on Day 28 (OR 0.52; 95% CI: 0.36–0.76 per unit increase; n = 91) after adjusting for hyperparasitemia, and age. Hyperparasitemia and age were not associated with anemia on Day 28 in the multivariable analysis. In the absence of a valid measure of total parasite burden (such as HRP2), the determination of peripheral parasitemia may have underestimated the impact of parasitemia.

**Table 2:** Risk factors for prolonged anemia at Day 14 (n = 92) and Day 28 (n = 91) calculated by logistic regression.

Risk factor	OR for anemia on Day 14	p-value	AOR for anemia on Day 14*	p-value	OR for anemia on Day 28	p-value	AOR for anemia on Day 28*	p-value
Hb Day 0, per unit increase	0.27 (0.16–0.45)	<0.001	0.27 (0.16–0.47)	<0.001	0.50 (0.35–0.72)	<0.001	0.52 (0.36–0.76)	0.001
Hyperparasitemia	10.00 (2.72–36.79)	0.001	8.76 (1.64–46.82)	0.011	2.98 (1.05–8.46)	0.040	1.43 (0.43–4.75)	0.559
Age, per year increase	0.84 (0.70–1.00)	0.047	0.98 (0.75–1.28)	0.872	0.84 (0.68–1.03)	0.091	0.92 (0.73–1.16)	0.464
Sex (boys compared to girls)	1.64 (0.71–3.79)	0.250	—	—	0.93 (0.35–2.47)	0.882	—	—

*AORs were adjusted for hemoglobin on Day 0, presence of hyperparasitemia on Day 0 and age.

No patient in the study had sickle cell anemia, of those with genotyping available (n = 108) only 4 were heterozygous for Hemoglobin S, 9 for hemoglobin C, and one had hemoglobin C disease. Due to the ensuing low power, hemoglobinopathies were not further assessed as risk factors for anemia. Similarly, the sample size was too low to detect any association between G6PD status in boys and anemia.

7 patients (5%) developed PADH, 5 of which have been described before^10^. The two new cases of PADH were only moderately affected with a nadir in hemoglobin of 10.0 g/dL in both patients on Day 14. For these two patients, pitting data were available. Compared to 17 children with a similar parasitemia range (50,000/µL to 110,000/µL) without PADH, there was some evidence that the median number of pitted erythrocytes was higher on Day 7 in children with PADH (78,269/µL vs 37,524/µL; p = 0.063) but not on Day 2 (69,068/µL vs 52,882/µL, p = 0.768). There was some evidence for a larger decrease in pitted erythrocytes between Days 7 and 14 in children with PADH (Δ pitted erythrocytes: 48,617/µL vs. 6,423/µL; p = 0.084).

**Figure 3:**
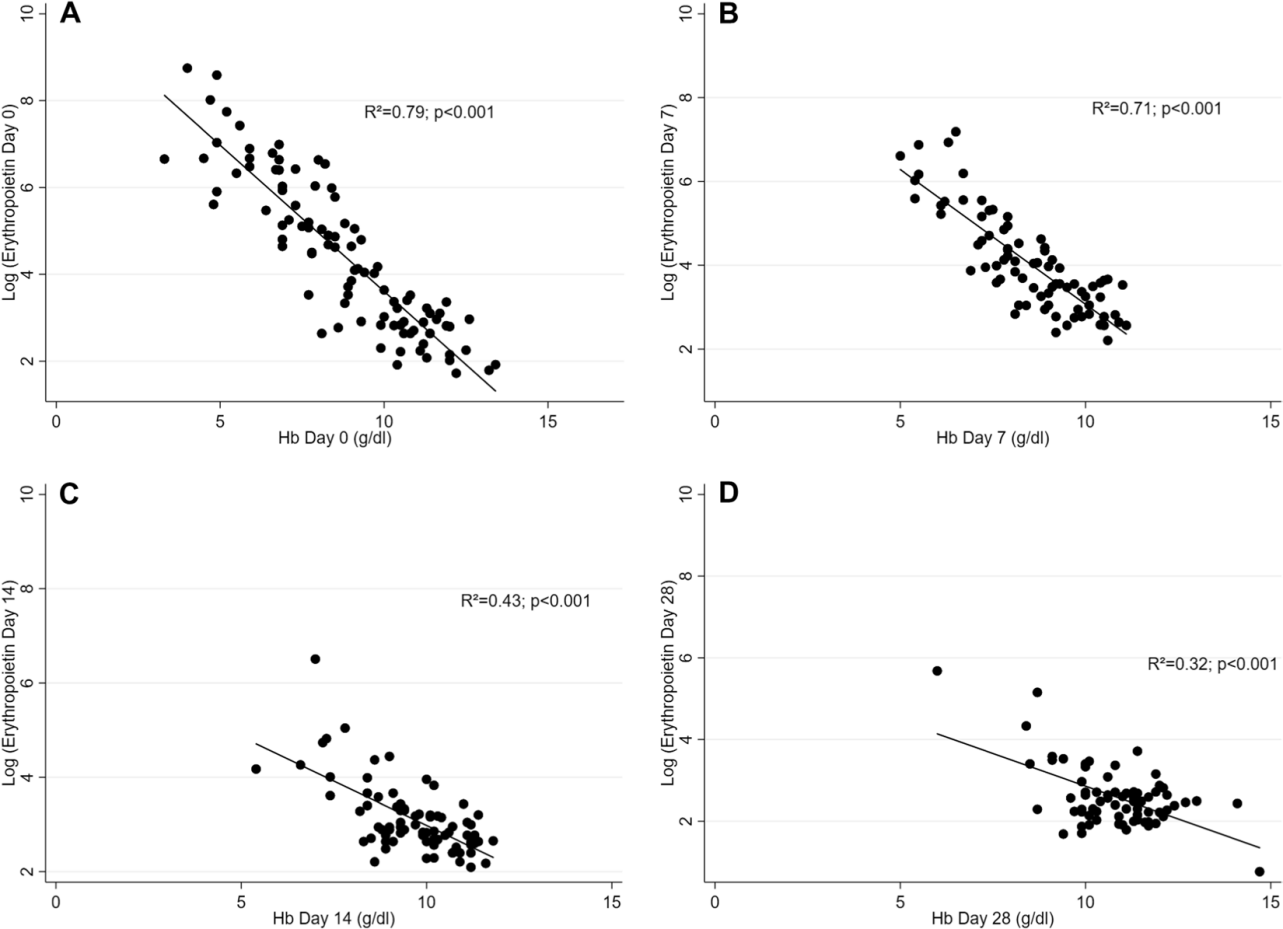
Association between hemoglobin levels and erythropoietin levels at different visits. (**A**) Visit Day 0: all children with measurements included (n = 96). (**B**) Visit Day 7: only non-transfused children (n = 82). (**C**) Visit Day 14: only non-transfused children (n = 81). (**D**) Visit Day 28: only non-transfused children (n = 78).

**Figure 4:**
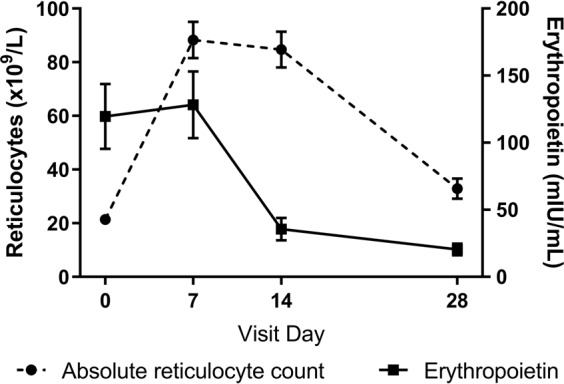
Absolute reticulocyte count and erythropoietin over time (mean ± SEM).

Figure 3 shows the association between hemoglobin levels and erythropoietin levels for the different visits. For analysis of Day 0 data, all patients with enough blood sample volume for erythropoietin measurements (n = 96) were included, for follow-up visits only those not having received blood transfusions were analyzed. On all days there is a strong log-linear association of erythropoietin with hemoglobin (p < 0.001), with hemoglobin explaining more than half of the variance of erythropoietin on Days 0 and 7 (R² = 0.79 and 0.71, respectively). Erythropoietin levels are highly elevated in response to severe anemia (Table 3). Reticulocytes show a time lag in the response to anemia, with reticulocytes count still being low on Day 0 while erythropoietin is already elevated at presentation (Fig. 4). After adjusting for hemoglobin at presentation, age, and hyperparasitemia, the logarithm of erythropoietin at presentation strongly predicts the increase in reticulocyte count between Day 0 and Day 7 (increase in the absolute reticulocyte count of 27.8 × 10^9^/L per unit increase in the logarithm of erythropoietin; 95% CI: 8.8–46.9; p = 0.005).

**Table 3:** Erythropoietin levels stratified by hemoglobin at presentation.

Hemoglobin at presentation (g/dL)	Erythropoietin (mIU/mL), median (range)
<5	963 (273–6297)
5–7.4	609 (104–2303)
7.5–9.9	88 (10–763)
≥10	16 (6–34)

## Discussion

In this population, children show a satisfactory hematological recovery after treatment with parenteral artesunate for malaria with a steady increase of hemoglobin after Day 7. The initial decline in hemoglobin up to the nadir on Day 7 is explained by the destruction of infected and uninfected red blood cells in acute malaria^25^. Additionally, malaria itself as well as the artemisinins impair erythropoiesis and early erythrocytic stages and reticulocytes are destroyed by erythrophagocytosis^14,26,27^. In combination with the potential destruction of infected reticulocytes, this translates to an inadequately low reticulocyte count at presentation, while erythropoietin is already elevated in response to anemia. There is a strong association between erythropoietin levels and hemoglobin levels at all time points, showing an adequate erythropoietin response. There are contradictory reports on whether the erythropoietin response is adequate in symptomatic malaria patients mainly due to methodological issues in older studies^28–30^. Our study showed an initially blunted response to erythropoietin but not a reduction in erythropoietin itself. Accordingly, after the initial lag phase, children showed a strong increase in reticulocytes and the erythropoietic response leads to a hematological recovery.

The major risk factor for prolonged anemia is a low hemoglobin level on presentation with an influence of baseline parasitemia on Day 14 but not on Day 28. Baseline causes of anemia, such as iron deficiency, malnutrition, hemoglobinopathies, and chronic infections prevail in malaria-endemic countries^12^. To explore the interplay of these covariates on malarial anemia and its recovery, larger cohort studies are needed. However, our data show that children recover from the early post-malarial decline in hemoglobin within one month after treatment. By raising the baseline hemoglobin through population-based measures, such as iron supplementation or mass treatment of helminthic infections, the duration and severity of malarial anemia on the individual child could be reduced. Similarly through reductions in malaria incidence and the subsequent reduction in malaria episodes per child, baseline hemoglobin values are likely to increase and the impact of each episode could be minimized^31^.

All children presenting with a hemoglobin level of less or equal than 5 g/dL received a transfusion, as WHO recommends^6^. Similarly, all children except one with a hemoglobin of less or equal than 6 g/dL have been transfused. There was no consensus for transfusing those children with a hemoglobin level between 6 and 7 g/dL, showing that the indication for transfusion in this group is mainly guided by individual patient factors. A conservative approach to transfusion has been historically propagated to ensure availability of blood transfusions and in the light of potential blood safety issues in African countries. However, there has been no solid evidence guiding transfusion thresholds in African children – especially not in severe malaria^32^. In our sample, transfusing children increased their hemoglobin up to 28 days after malaria compared to those without transfusion with the same starting hemoglobin. Whether this increase in hemoglobin also directly or indirectly reduces morbidity (such as fatigue or susceptibility to infections) is unclear. A recent randomized controlled trial in African children (of which 63% had malaria) did not show a difference in clinical outcomes after 6 months between aggressive and conservative transfusion approaches^33,34^.

In addition to five previously reported patients with PADH, we identified two additional patients with signs of PADH in the follow-up study^10^. The overall prevalence and severity of PADH seems to be lower in patients living in malaria-endemic settings than in returning European travelers with severe malaria^4,5,11^. Available evidence supports an overall frequency of PADH in African children treated with artesunate of 5% - with severe PADH occurring in 1% of children^10,11,35^. While these results provide some reassurance, an adverse reaction with a frequency of 1% would still be defined as “common” by the standard categories as recommended by the Council for International Organizations of Medical Sciences (CIOMS)^36^. Children reported in our studies had only moderately severe malaria; only a small proportion had very high parasite counts. We cannot rule out a significantly higher frequency of PADH in children with parasite counts over 250,000/µL. More evidence in this patient population with high parasitemias is critical, especially as the life-saving benefit of artesunate is highest in this patient group^1^.

With a lower than expected incidence of PADH (linked to lower than expected parasite counts), pathophysiological analyses can only remain exploratory due to a very low sample size.

## Conclusion

Most children with malaria show an adequate erythropoietic response to malarial anemia and show a full recovery one month after treatment with parenteral artesunate. The incidence of PADH was lower than expected, but it should be investigated as a risk factor for anemia in a higher risk population with higher parasitemia. The main risk factor for prolonged anemia in our cohort of African children treated with artesunate is pre-existing anemia upon presentation.

## Supplementary information

Supplementary information

## Data Availability

The datasets analyzed during the current study are not publicly available due to them containing information that could compromise research participant privacy/consent but are available from the corresponding author on reasonable request.
