# Supplementary material for: Determinants of post-malarial anemia in African children treated with parenteral artesunate
Source: Sci Rep. 2019 Dec 2;9:18134. doi: 10.1038/s41598-019-54639-4 (PMC6888809; doi:10.1038/s41598-019-54639-4)
Supplement: Supplementary file 1 — Supplementary information [file 41598_2019_54639_MOESM1_ESM.pdf]

Determinants of post-malarial anemia in African children treated with parenteral artesunate

Authors: Katrin Scheu, Ayola Akim Adegnika, Marylyn M. Addo, Daniel Ansong, Jakob P. Cramer, Svenja Fürst, Peter G. Kremsner, Florian Kurth, Thomas Jacobs, Jürgen May, Michael Ramharter, Justice Sylverken, Christof D. Vinnemeier, Tsiri Agbenyega, Thierry Rolling

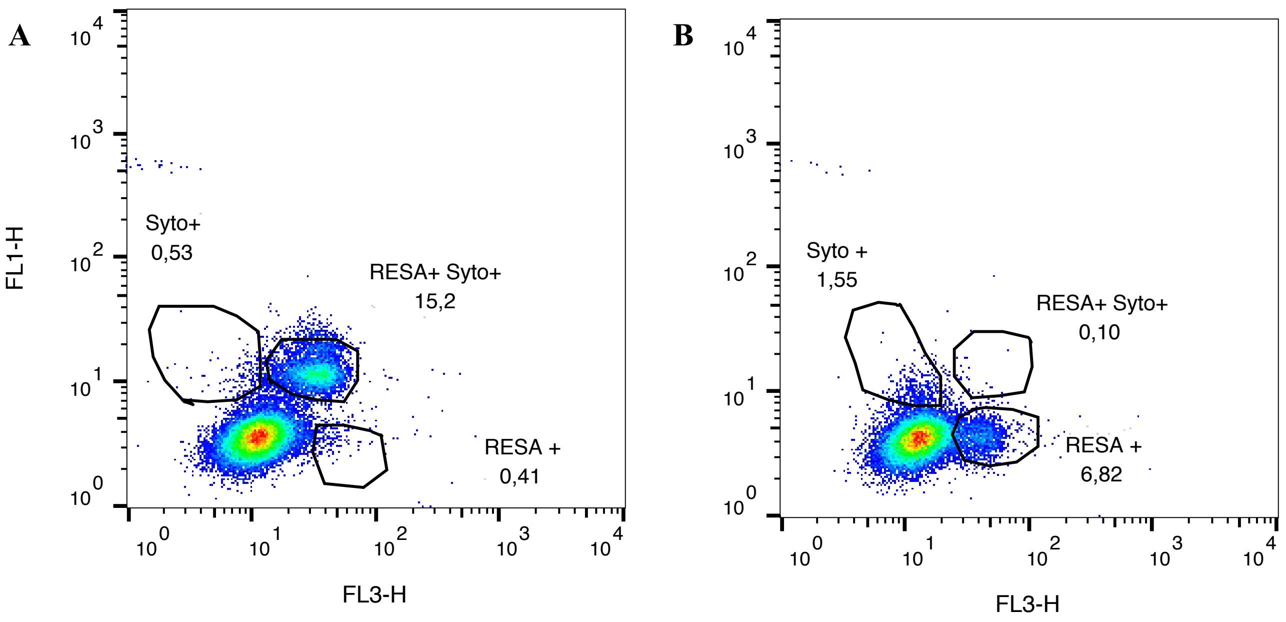

Supplementary Figure 1: Representative flow cytometry plots. A) Sample from Day 0 with 15.2% infected RBCs (RESA+Syto+). B) Sample from the same patient from Day 2 with 6.82% once-infected RBCs (RESA+Syto-).
